# 1177. Clinical Pharmacist-Lead Amoxicillin Oral Challenges in Low-Risk Penicillin Allergy Patients

**DOI:** 10.1093/ofid/ofad500.1017

**Published:** 2023-11-27

**Authors:** Breana Caturano, Lauren Bjork, Viviana Temino

**Affiliations:** Miami VA, Miami, Florida; Miami VA Medical Center, Miami, Florida; Miami VA, Miami, Florida

## Abstract

**Background:**

Amoxicillin direct oral challenge (DOC) without preceding skin testing in patients with low-risk penicillin (PCN) allergy histories has been shown to be a safe and effective method to delabel PCN allergies. The implementation of robust DOC programs is dependent on Allergy/Immunology oversight. Prior to this initiative, our institution primarily offered PCN skin testing to outpatients and DOC was limited. To expand upon our services, a clinical pharmacist was trained to independently interview, risk-stratify, and perform DOC in patients with low-risk PCN allergy histories.

**Methods:**

Adult inpatients with a documented PCN allergy were evaluated by a clinical pharmacist based on the local algorithm created by Allergy (Figure 1). Patients who met criteria were offered amoxicillin 500 mg PO and observed for 60 minutes. Epinephrine and diphenhydramine were available in case of adverse reaction. If no reaction resulted, the PCN allergy label was removed.

**Figure 1.**
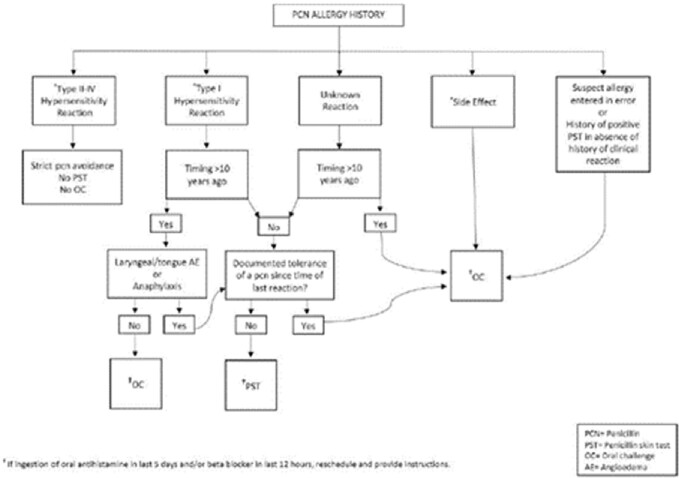
Penicillin Allergy History Evaluation Algorithm

Adult inpatients with a documented PCN allergy were evaluated by a clinical pharmacist based on the local algorithm created by Allergy Service.

**Results:**

A total of 136 patients were screened from October 2021-March 2023 with a mean age of 67 and 89% were male (Table 1). The most common reaction was unknown (Table 2). Of the patients screened, 20% did not qualify for DOC and 15% were delabeled based on evaluation. During the study period, 65% of patients screened were qualified for DOC with 26% that received DOC. None of the DOC patients had an immediate adverse reaction, although one patient experienced a delayed reaction. Limitations included 34 clinically unstable patients and 22 DOC patients deferred and lost to follow-up outpatient (Figure 2).

**Figure:**
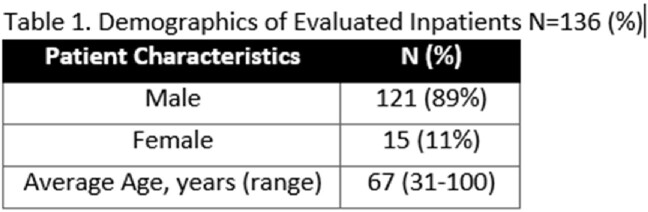

**Figure:**
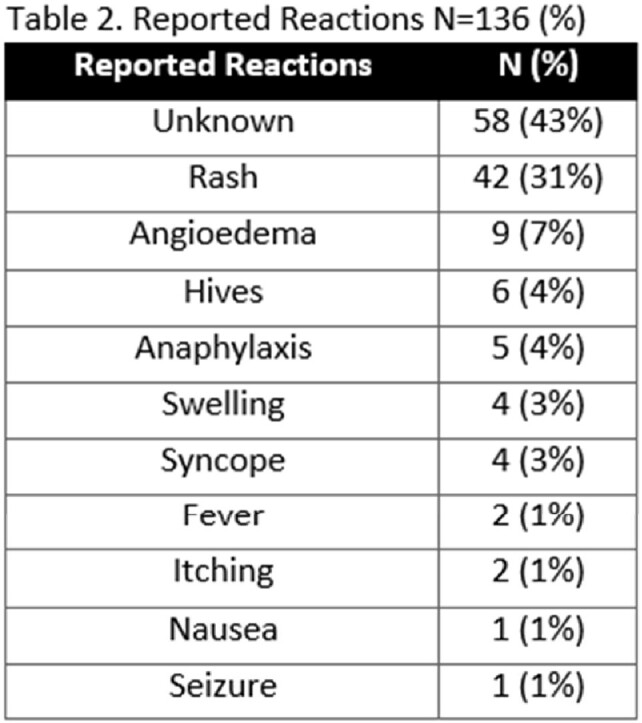

**Figure:**
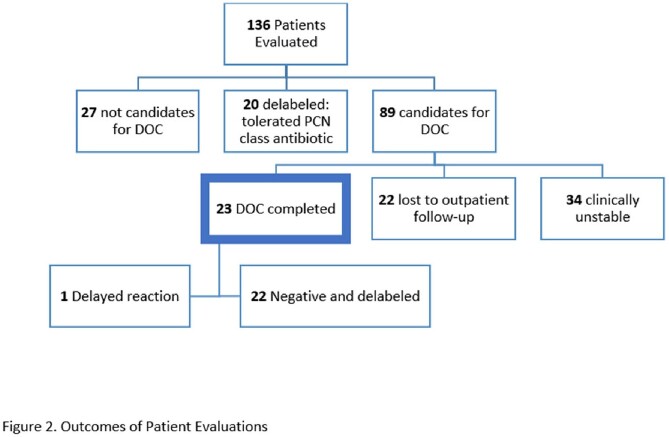

**Conclusion:**

Expanding the scope of practice of a clinical pharmacist to perform evaluations, risk assessments, and DOC on inpatients is a safe and effective method for those with low-risk PCN allergy histories. Institutions with adult patients may consider implementation of clinical pharmacist-led allergy delabeling program to expand upon current practices.

**Disclosures:**

**All Authors**: No reported disclosures

